# Shiga Toxin Induces Apoptosis *via* ROS–Caspase Activation in Human Cerebral Endothelial Cell Line hCMEC/D3 and Astrocyte Co-Culture

**DOI:** 10.4014/jmb.2512.12006

**Published:** 2026-01-08

**Authors:** Mirim Kim, Kyung-Soo Lee, Jun Young Park, Chang-Ung Kim, Yu-Jin Jeong, Moo-Seung Lee

**Affiliations:** 1 Environmental Diseases Research Center, Korea Research Institute of Bioscience and Biotechnology, 125 Gwahak-ro, Yuseong-Gu, Daejeon 34141, Republic of Korea; 2 Department of Biomolecular Science, KRIBB School of Bioscience, Korea University of Science and Technology (UST), 127 Gajeong-ro, Yuseong-gu, Daejeon 34113, Republic of Korea; 3 College of Pharmacy, Chosun University, Gwangju 61452, Republic of Korea; 4 Research Institute of Pharmaceutical Sciences (RIPS), College of Pharmacy, Chosun University, Gwangju 61452, Republic of Korea

**Keywords:** Shiga toxin, Hemolytic uremic syndrome, Microvascular endothelial cell, Apoptosis, EHEC

## Abstract

Hemolytic uremic syndrome (HUS), a fatal complication of Shiga toxin-producing *Escherichia coli* (STEC) infection, is classically characterized by acute renal failure, but frequently accompanied by central nervous system (CNS) dysfunction. Because the CNS is normally protected by the blood-brain barrier (BBB), the toxin-mediated BBB injury is considered to be a major cause of neurologic sequelae in STEC infection. Here, we delineate how Shiga toxin type 1a (Stx1a) and Shiga toxin type 2a (Stx2a) compromise BBB-like endothelial barrier integrity with the human brain microvascular endothelial cell line hCMEC/D3 as an *in vitro* model, complemented by an endothelial–astroglial co-culture system. Stx1a/Stx2a exposure induced the MAPK pathway and ER stress, triggering caspase-mediated apoptosis and pro-inflammatory cytokines expression. Coincidentally, permeability across tight junctions was impaired, with junctional protein loss and increased paracellular permeability. Pharmacologic inhibition of caspases prevented cytotoxicity and tight junction loss, indicating a role for the apoptotic process in barrier breach. In co-cultures with transwell, human astrocytes (A172) demonstrated caspase activity and cytokine induction even without direct exposure to toxins, indicating endothelial injury–release paracrine activity in the propagation of BBB injury. Reactive oxygen species (ROS) also accumulated distal to toxin exposure and aligned with apoptotic and barrier phenotypes, indicating a ROS–caspase pathway in endothelial cell injury. Collectively, our findings show that Stx may impair BBB integrity through ROS accumulation and caspase-dependent apoptosis. This is the first study establishing hCMEC/D3 cells as a model for elucidating Stx-induced BBB disruption, providing mechanistic insights for the therapeutic development against CNS complications in HUS.

## Introduction

Hemolytic uremic syndrome (HUS) is a fatal, toxin-mediated microangiopathy predominantly manifested as an acute renal failure that is disproportionately prevalent in children and the elderly, typically after the ingestion of contaminated meat, water, or unpasteurized milk [1, 2]. The principal perpetrators responsible for HUS are Shiga toxins (Stxs), AB5 protein exotoxins produced by Shiga toxin–producing *Escherichia coli* (STEC) and *Shigella dysenteriae* serotype 1. Globally, foodborne STEC infections have a substantial public health impact, with the World Health Organization estimating over 1 million illnesses and roughly 13,000 disability-adjusted life years in 2020 [3]. Stxs comprise multiple serological variants classified under two main families (Stx1 and Stx2), with clinically significant subtypes such as Stx1a and Stx2a [4]. Structurally, Stxs are assembled as an AB5 holotoxin: a single A subunit noncovalently associated with five identical B subunits forming a pentameric ring [5]. The A subunit possesses *N-glycosidase* activity that selectively depurinates a single adenine residue (A4324) within 28S rRNA, thereby halting protein synthesis and activating cellular apoptosis [6]. The B pentamer binds the glycosphingolipid globotriaosylceramide (Gb3) present on host-cell membranes, mediating endocytosis and retrograde trafficking to the Golgi and endoplasmic reticulum (ER) while evading lysosomal degradation, ultimately leading to cytotoxicity [7].

Although renal injury is the defining clinical feature of HUS, central nervous system (CNS) involvement including seizures, stroke, encephalopathy/coma, visual loss, and other neurologic symptoms occur in a substantial proportion of patients and is associated with adverse outcomes [8-10]. In a 33-year pediatric cohort, neurological manifestations ranged from seizures (71%) and altered consciousness (85%) to focal weakness/paralysis (40%) [8]. The CNS is typically shielded by the blood–brain barrier (BBB), a neurovascular unit composed of brain microvascular endothelial cells, pericytes, basement membrane, and astrocytic endfeet, that tightly regulates paracellular and transcellular transport of solutes and cells [11]. The selectivity of BBB is regulated mostly by the junctional complex between endothelial cells—tight junctions (TJs), adherent junctions, and gap junctions—with TJs (*e.g.*, claudins, occludin, and ZO proteins) forming a tightly selective diffusion barrier that maintains endothelial polarity and facilitates intracellular signaling [12, 13]. This stringent barrier is also the principal reason that >98% of small-molecule drugs and nearly all biologics are ineffective in accessing the brain parenchyma [14]. Despite this, clinical observations indicate that Stxs are capable of permeating BBB barriers and causing CNS pathology. Previous studies have shown that Stxs can damage astrocytes and other neural components, yet the endothelial-centric, BBB-resolving processes, injury biomarkers, and paracrine propagation of damage remain incompletely defined [15]. In an effort to fill this gap, we employed the human brain microvascular endothelial cell line hCMEC/D3 as a tractable *in-vitro* BBB endothelium and questioned cellular and molecular responses to Stx1a and Stx2a. hCMEC/D3 cells express tight junction proteins as well as multiple transporters and efflux pumps and form a low-permeability monolayer, making them a suitable *in vitro* model for evaluating changes in blood–brain barrier integrity and permeability [16, 17]. We determined that exposure to Stx activates stress-activated MAPK cascades and ER stress, with resultant caspase-dependent apoptosis and a pro-inflammatory transcriptional profile. Coinciding with the disruption of endothelial TJs, with loss of junctional proteins and increased paracellular permeability, such events provide a direct mechanistic link between toxin signaling and failure of the barrier. Functionally, inhibition of caspase activity attenuated cytotoxicity and maintained expression of TJ proteins, implicating the apoptotic pathway as a critical driver of BBB dysfunction. Recognizing that barrier physiology is an emergent property of the neurovascular unit, we extended our analysis to a transwell co-culture of hCMEC/D3 endothelial cells with human astrocytes (A172), better approximating the BBB microenvironment [18, 19]. Notably, astrocytes displayed caspase activation and cytokine induction independent of direct toxin exposure, indicating paracrine endothelial–astroglial signaling as a conduit of injury propagation across the BBB interface. Overall, these studies support a model in which Stx1a/Stx2a compromise BBB integrity via ROS accumulation, MAPK/ER stress signaling, and caspase-executed apoptosis, crosstalk-signaling between endothelial cells and astrocytes may further augment neurovascular injury. By discerning these endothelial-centric mechanisms and their downstream consequences, our work provides mechanistic insight into Stx-mediated BBB dysfunction and a rationale for therapeutic interventions that target oxidative stress, ER stress responses, or apoptotic execution to mitigate CNS complications in HUS.

## Materials and Methods

### Toxins

Stx2a purified from *Escherichia coli* was purchased from List Biological Laboratories, Inc. (USA). Stx1-B subunit was purchased from Sigma (Merck, Germany). Stx2B was purchased from Creative Diagnostics (USA). Stx1a and Stx2a mutant containing the triple mutations Y77S/E167Q/R170L in the enzymatic active site of the A subunit were a generous gift from Professor Vernon L. Tesh, Texas A&M University, USA.Stx1a was purified in the laboratory of Professor Vernon L. Tesh from recombinant Stx1a-expressing *E. coli* DH5α (pCKS112) using sequential ion exchange and immunoaffinity chromatography. Purity was confirmed by SDS-PAGE, silver staining, and Western blot analysis. Endotoxin contaminant levels were reduced to <0.1 ng/ml using an ActiClean Etox column (Sterogene Bioseparations, USA). Residual endotoxin contamination was assessed using the Limulus Amoebocyte lysate assay (Associates of Cape Cod, USA). A purified Stx1a mutant containing double mutations (E167Q and R170L) in the A subunit was a generous gift from Dr. Shinji Yamasaki, Osaka Prefecture University, Japan.

### Cell Culture

The human brain endothelial cell line hCMEC/D3 (Merck) was cultured in Endothelial Cell Growth Medium (PromoCell, Germany) supplemented with 1% Antibiotic-Antimycotic (Thermo Fisher Scientific, USA), and human astrocyte cell line A172 was cultured in RPMI 1640 medium (Corning, Thermo Fisher Scientific) supplemented with 10% FBS and 1% Antibiotic-Antimycotic (Thermo Fisher Scientific) at 37°C in a humidified incubator containing 5% CO_2_. hCMEC/D3 and A172 cells were seeded at 5.0 × 10^5^ cells/well into 6-well plates, washed once with sterile Dulbecco's phosphate-buffered saline (DPBS) (Sigma-Aldrich, USA), and treated with 10 ng/ml Stx2a in Endothelial Cell Growth Medium or RPMI containing 0.5% FBS without supplements for the indicated time periods.

For the co-culture of hCMEC/D3 and A172 cells, hCMEC/D3 cells were seeded at 3.0 × 10^5^ cells/well in insert of a trans-well (Corning, Thermo Fisher Scientific, USA), and A172 cells were seeded at 5.0 × 10^5^ cells/well in the lower well. After washing once with sterile Dulbecco’s phosphate-buffered saline (DPBS) (Sigma-Aldrich), cells were treated with 10 ng/ml Stx2a for various times in Endothelial Cell Growth Medium and RPMI containing 0.5% FBS and without supplements in all experiments.

### Cytotoxicity Assay

Cell supernatants were used to determine cytotoxicity by measuring lactate dehydrogenase (LDH) release. Experiments were performed using the Pierce LDH Cytotoxicity Assay Kit according to the manufacturer's instructions. Absorbance was measured at 490 nm and 680 nm using a SpectraMAX 190 Microplate Reader (Molecular Devices, USA). The background concentration was removed by subtracting the 680 nm absorbance of each sample from the 490 nm absorbance. Cell death was assessed using the Muse Annexin V & Dead Cell Kit (Cytek Biosciences, USA) according to the manufacturer's protocol. The WST-8 dye-based assay was performed using the WST-8 Cell Counting Kit (QM1000) (Biomax, Republic of Korea) according to the manufacturer's protocol. Briefly, one-tenth of the cultured hCMEC/D3 cell suspension treated with Stx2 was incubated with WST-8 reagent at RT for an appropriate amount of time. The incubated culture medium was then transferred to a 96-well plate in triplicate, and the absorbance was measured at 450 nm using a SpectraMAX 190 microplate reader (Molecular Devices, USA).

### Flow Cytometry Analysis to Confirm Gb3 (CD77) Expression

hCMEC/D3 cells were seeded in 6-well plates at 5.0 × 10^5^ cells/well and cultured in Endothelial Cell Growth Medium (PromoCell) containing 1% Antibiotic-Antimycotic (Thermo Fisher Scientific). Cells were maintained at 37°C in a humidified incubator containing 5% CO_2_. After 24 h, hCMEC/D3 cells were washed three times with cold PBS, hCMEC/D3 cells were detached with trypsin-EDTA (Gibco, Thermo Fisher Scientific) and collected by centrifugation at 780 × *g* for 5 min. Cells were then stained with Alexa Fluor 647-conjugated anti-human Gb3/CD77 monoclonal antibody (BD Biosciences, USA) or Alexa Fluor 647-conjugated mouse IgM (isotype control) for 30 min at 4°C in the dark and analyzed by flow cytometry using a BD FACS Canto ІІ cell analyzer (BD Bioscience) [10].

### Caspase-3/7 Activity Analysis

hCMEC/D3 cells were seeded in 6-well plates at 5.0 × 10^5^ cells/well and cultured in Endothelial Cell Growth Medium (PromoCell) containing 1% Antibiotic-Antimycotic (Thermo Fisher Scientific). Cells were maintained at 37°C in a humidified incubator containing 5% CO_2_. Cells were pretreated with z-VAD (Selleckchem, USA) for 1h before exposure to Stx2a (10 ng/ml). After 24 h, the culture medium was removed, and 100 μl of the supernatant was returned to the wells. Then, the cells were incubated with 100 μl of Caspase-Glo 3/7 reagent (Promega, USA) containing substrate for 1–3 h. Next, the reaction mixture was transferred in triplicate to a 96-well Flat Clear bottom white microplate (Corning, Thermo Fisher Scientific) and luminescence was measured using a VICTOR Nivo Microplate Reader (PerkinElmer, USA).

### Western Blot Analysis and Antibodies

All cells were harvested at 1h intervals and lysed with CETi lysis buffer (TransLab, Republic of Korea). Protein concentration was measured using the Pierce BCA protein assay kit (Thermo Fisher Scientific). Equal amounts of protein samples (20–40 μg/lane) were dispensed onto Bolt Bis-Tris Plus 4–12% gradient gels (Thermo Fisher Scientific) and transferred to polyvinylidene fluoride (PVDF) membranes via electrophoresis at 180 V. Membranes were blocked with PVDF Blocking Reagent (TOYOBO, Japan) for 1 h, followed by three 5-min washes with TBST (20 mM Tris [pH 7.6], 137 mM NaCl, 0.1% Tween 20). The PVDF membranes were then incubated overnight at 4°C with the appropriate primary antibody. After incubation, the PVDF membranes were washed three times with TBST and incubated with horseradish peroxidase (HRP)-conjugated secondary antibodies for 90 min at room temperature (21–23°C) in the dark. Band detection was performed using the Immobilon Forte Western HRP substrate (Merck) and Odyssey Scanner (LI-COR, USA). The antibodies used were as follows:

Primary antibodies: PERK, Phospho-PERK, IRE1α, eIF2α, Phospho-eIF2α, Bip, Cleaved Caspase-3, Caspase-3, p-P38, P38, p-JNK, JNK, P-ERK, ERK, Claudin-1, ZO-1, E-cadherin (Cell Signaling Technology, USA), phospho-IRE1α (Abcam, UK). HRP-conjugated human β-actin-specific monoclonal antibody (Cell Signaling Technology). Secondary antibody: HRP-conjugated anti-rabbit IgG (Cell Signaling Technology)

### Intracellular Trafficking Assay

Cell culture slides (SPL Life Sciences, Republic of Korea) were coated with collagen type 1, rat tail (Merck), and hCMEC/D3 cells were seeded at 1.0 × 10^5^ cells/well. Alexa Fluor 488-conjugated Stx1a (100 ng/ml) was treated for 1 h and 30 min. Cells were washed with DPBS (Sigma-Aldrich), and the cells were fixed by treating with 1 ml of 4% formaldehyde (Biosolution, Republic of Korea) for 2 min. Next, wash the formaldehyde with DPBS and add ER-Tracker Red (BODIPY TR Glibenclamide) (Invitrogen, Thermo Fisher Scientific) and NucBlue Fixed Cell ReadyProbes Reagent (DAPI) (Invitrogen, Thermo Fisher Scientific, USA) and incubate for 20 min at 37°C in a humidified 5% CO_2_ environment. Afterwards, the cells were washed, covered with cover glass, and mounted with Mountant with NucBlue (Invitrogen, Thermo Fisher Scientific). Using a fluorescence microscope EVOS M5000 (Thermo Fisher Scientific) with fluorescence signals detected in red, green, and blue emission channels.

### Reverse Transcription - Quantitative PCR

Total RNA was extracted using NucleoSpin RNA Plus (Macherey-Nagel, Germany). cDNA synthesis and PCR amplification using reverse transcriptase were performed using the NanoHelix RT-qPCR kit (NanoHelix, Republic of Korea) according to the manufacturer's protocol. The cycling conditions for real-time PCR were as follows: 40 min at 50°C, followed by 15 min of PCR enzyme activation at 95°C. This was followed by 40 cycles of 20 s at 95°C, 30 sec at each primer annealing temperature, and 1 min at 72°C. Fluorescence data were collected using SYBR Green with a qTOWER³ (Analytik Jena, Germany). All mRNA expression data were normalized to GAPDH expression. Primer sequences are listed below (Table 1).

### NO Assay

Collect the supernatant from hCMEC/D3 cells treated with Stx2a (10 ng/ml) and NAC (MedChemExpress, USA). Use the nitrite standard solution included in the Griess Reagent Kit (Invitrogen, ThermoFisher Scientific) to create a standard curve. Supernatants were transferred in triplicate. Mix N-(1-naphthyl) ethylenediamine dihydrochloride (Component A) and Sulfanilic acid (Component B) in a 1:1 ratio and add to each well of a 96-well plate. After 30 min at room temperature, measure the absorbance at 548 nm using a SpectraMax190 microplate reader.

### ELISA

The concentrations of human IL-1β, IL-6, IL-8, CCL-2, and TNF-α, which are secreted cytokines and chemokines in the culture medium, were measured using ELISA kits (Invitrogen, ThermoFisher Scientific) according to the manufacturer's instructions. Cell supernatants were collected from hCMEC/D3 or A172 cells in a co-culture model treated with Stx2a (10 ng/ml). Briefly, 50 μl of each capture antibody solution was coated on 96-well half microplates and incubated overnight at 4°C. The next day, wash the wells once with PBS-T (10 mM phosphate buffer, 2.7 mM KCl, 137 mM NaCl, and 0.05% Tween 20, [pH 7.4]) and add 100 μl of 5X ELISA buffer diluted to 1X to each well and block for 1 h at room temperature. After 1 h, wash the wells three times, add 50 μl of each sample, and incubate for 2 h at room temperature. Wash the wells three times as described above, add 50 μl of each detection antibody, and incubate for 1 h at room temperature. Next, wash the wells three times, add 50 μl of streptavidin-HRP, and incubate for 30 min at room temperature, protected from light. Finally, wash the wells five times, add 50 μl of substrate solution, and incubate for 15 min, away from light. When a color change occurs, stop the reaction by adding 25 μl of 2 M H_2_SO_4_, and measure the absorbance at 450 nm using a SpectraMax190 microplate reader (Molecular Devices, USA).

### Cell-to-Cell Permeability Analysis

hCMEC/D3 cells were seeded at 3.0 × 10^5^ cells/well in the insert chambers of trans-well plates. Cells were treated with Stx1a (100 ng/ml), Stx1a^mut^ (100 ng/ml), Stx2a (10 ng/ml), or Stx2a^mut^ (10 ng/ml) for 24 h. Fluorescein-conjugated ovalbumin (Invitrogen, ThermoFisher Scientific), 45 kDa, was added to the insert wells containing toxin-treated hCMEC/D3 cells at the concentration recommended by the manufacturer. After 1 h, the supernatant was collected from the bottom well of the trans-well. The supernatant was transferred to a 96-well plate in triplicate (100 μl per well), and fluorescence was measured using a VICTOR Nivo Microplate Reader (PerkinElmer). The measured fluorescence values were used to evaluate cell permeability in response to each toxin.

### Quantitative Analysis

Image quantification was performed using ImageJ/Fiji. The Pearson's correlation coefficients of multiple image sets were calculated using the 'Colocalization' plugin in ImageJ/Fiji. The values ranged between 0 and 1; a value of 1 indicated complete co-localization, while a value of 0 indicated no co-localization.

### Statistical Analysis

Data were expressed as the means ± standard error of the mean (SEM). Group differences were evaluated by one-way ANOVA followed by Tukey post test, using GraphPad Prism version 5.00. (GraphPad Software, USA). A *p*-value < 0.05 was considered statistically significant. (* = *p* < 0.05; ** = *p* < 0.01; *** = *p* < 0.001).

## Result

### hCMEC/D3 Cells Undergo Cytotoxicity and Apoptosis Exposure to Stx1a and Stx2a

**Fig. 1:**
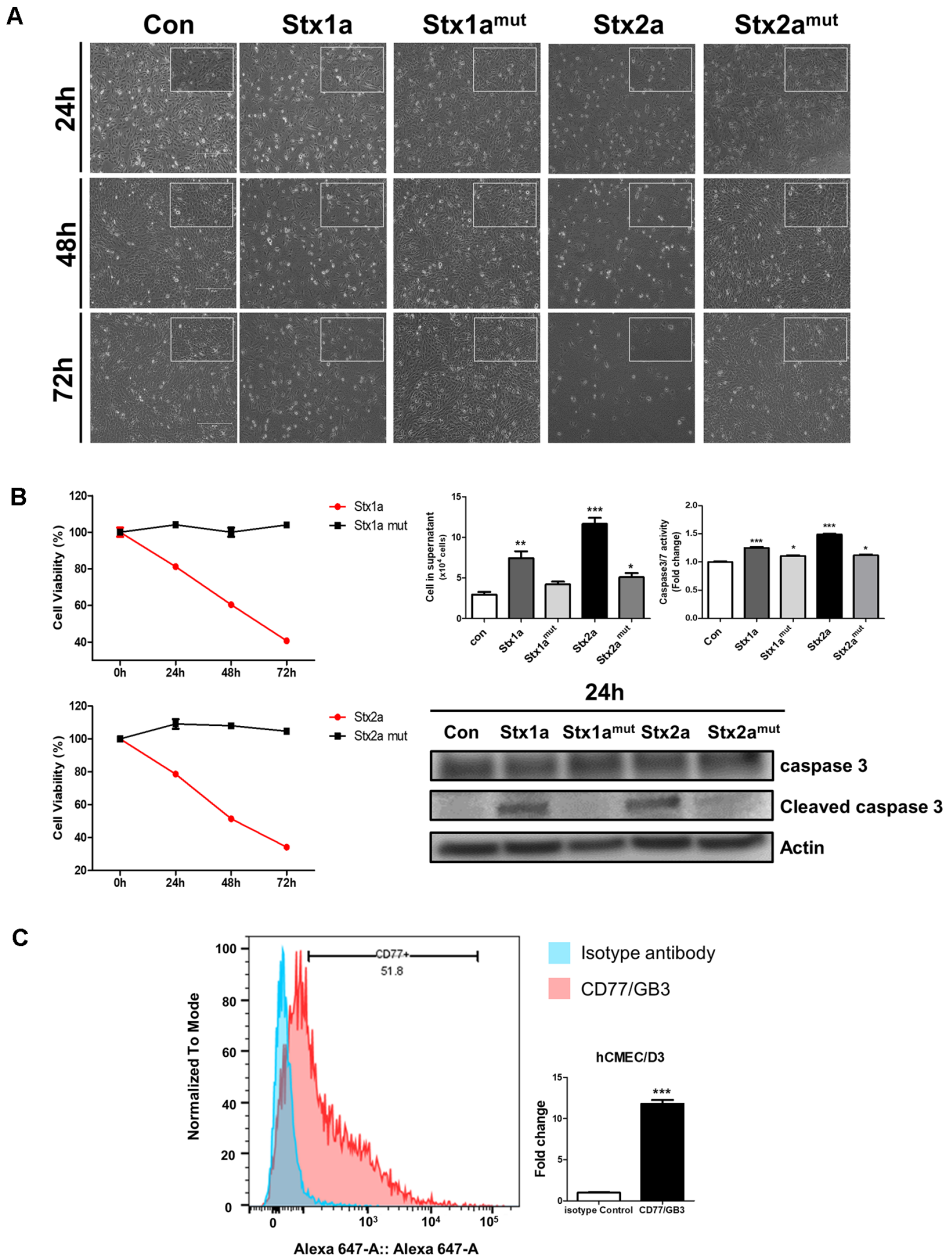
Effect of Stxs on apoptosis in hCMEC/D3 cells. (**A**) hCMEC/D3 cells treated with Stx1a or Stx2a exhibited prominent morphological alterations when observed under a fluorescence microscope at 20X magnification, whereas cells treated with Stx1a^mut^ or Stx2a^mut^ did not show such changes. Images were collected from cells incubated with or without Stxs for 24, 48, and 72 h; (**B**) hCMEC/D3 cells were seeded in 6-well plates (5.0 ×10^5^ cells/well) and incubated with Stx1a (100 ng/ml), Stx1a^mut^ (100 ng/ml), Stx2a (10 ng/ml) or Stx2a^mut^ (10 ng/ml) for 24, 48, 72 h. Cell viability was determined using WST-8 assays, expressed as percentage viability and fold change relative to untreated controls (*left panel*). hCMEC/D3 cells (1.0 × 10^5^ cells/well) were treated with Stx1a (100 ng/ml), Stx1a^mut^ (100 ng/ml), Stx2a (10 ng/ml) and Stx2a^mut^ (10 ng/ml) for 24 h, and caspase-3/7 activity was measured using the caspase Glo-3/7 assay. Under the same conditions, hCMEC/D3 cells (5.0 × 10^5^ cells/well) were treated with Stx1a (100 ng/ml), Stx1a^mut^ (100 ng/ml), Stx2a (10 ng/ml) and Stx2a^mut^ (10 ng/ml) for 24 h, and then protein samples were subjected to Western blotting using an anti-cleaved caspase-3 antibody (*right panel*). β-Actin was used as a control for equal protein loading. The results are a representative experiment obtained from three independent experiments; (**C**) Gb3 expression in hCMEC/D3 cells was detected by staining with Alexa Fluor 647-conjugated anti-CD77/Gb3 antibody or isotype control (mouse IgM-Alexa Fluor 647) for 1 h at 4oC. Representative results from three independent experiments are shown. Statistical significance. Asterisks indicate significant differences between control cell values and Stxs-treated cells (Panel B). *p < 0.05, **p < 0.01, ***p < 0.001 versus control.

To determine the effect of Stxs on hCMEC/D3 cells, we treated monolayers with Stx2a (10 ng/ml), catalytically inactive Stx2a^mut^ (10 ng/ml), Stx1a (100 ng/ml), and Stx1a^mut^ (100 ng/ml) at different time points [10, 20] and examined the cellular morphological characteristics of hCMEC/D3 cells (Fig. 1A). The concentrations of Stx1a and Stx2a were selected to induce comparable levels of cellular responses in hCMEC/D3 cells, reflecting previous studies demonstrating that Stx2a exhibits significantly higher biological activity and toxicity than Stx1a [10, 20]. hCMEC/D3 cells showed a typical spread morphology with extended processes in the control group, whereas cells treated with Stx1a and Stx2a displayed time-dependent cytopathic morphological changes, including intercellular gap widening and loss of cellular extensions. In contrast, cells treated with the Stx2a^mut^ and Stx1a^mut^ lacking the *N-glycosidase* activity of the A subunit exhibited morphological characteristics similar to those of the control cells. A cytotoxic assay (WST) on hCMEC/D3 cells exhibited a significant reduction in Stx1a and Stx2a-treated cells (Fig. 1B). The expression of Gb3, an essential receptor for Stx binding and endocytosis, was measured by fluorescence-activated cell sorting (FACS). Cultured hCMEC/D3 cells showed Gb3 expression (Fig. 1C, Fig. S1). Moreover, to assess apoptotic execution in Stxs-treated hCMEC/D3 cells expressing Gb3, we measured caspase-3/7 activity and the expression of immunoblotted cleaved caspase-3, a key protein mediating apoptosis (Fig. 1B). We confirmed that cleaved caspase-3 expression was significantly increased in cells treated with Stx1a and Stx2a, and that caspase-3/7 activity was also increased. These results indicate that hCMEC/D3 cells are sensitive to Stxs-induced cytotoxicity, supporting caspase-mediated apoptosis as a principal mode of Stx cytotoxicity in hCMEC/D3.

### Intracellular Translocation of Stxs Induces ER Stress and ROS Production in hCMEC/D3 Cells

**Fig. 2:**
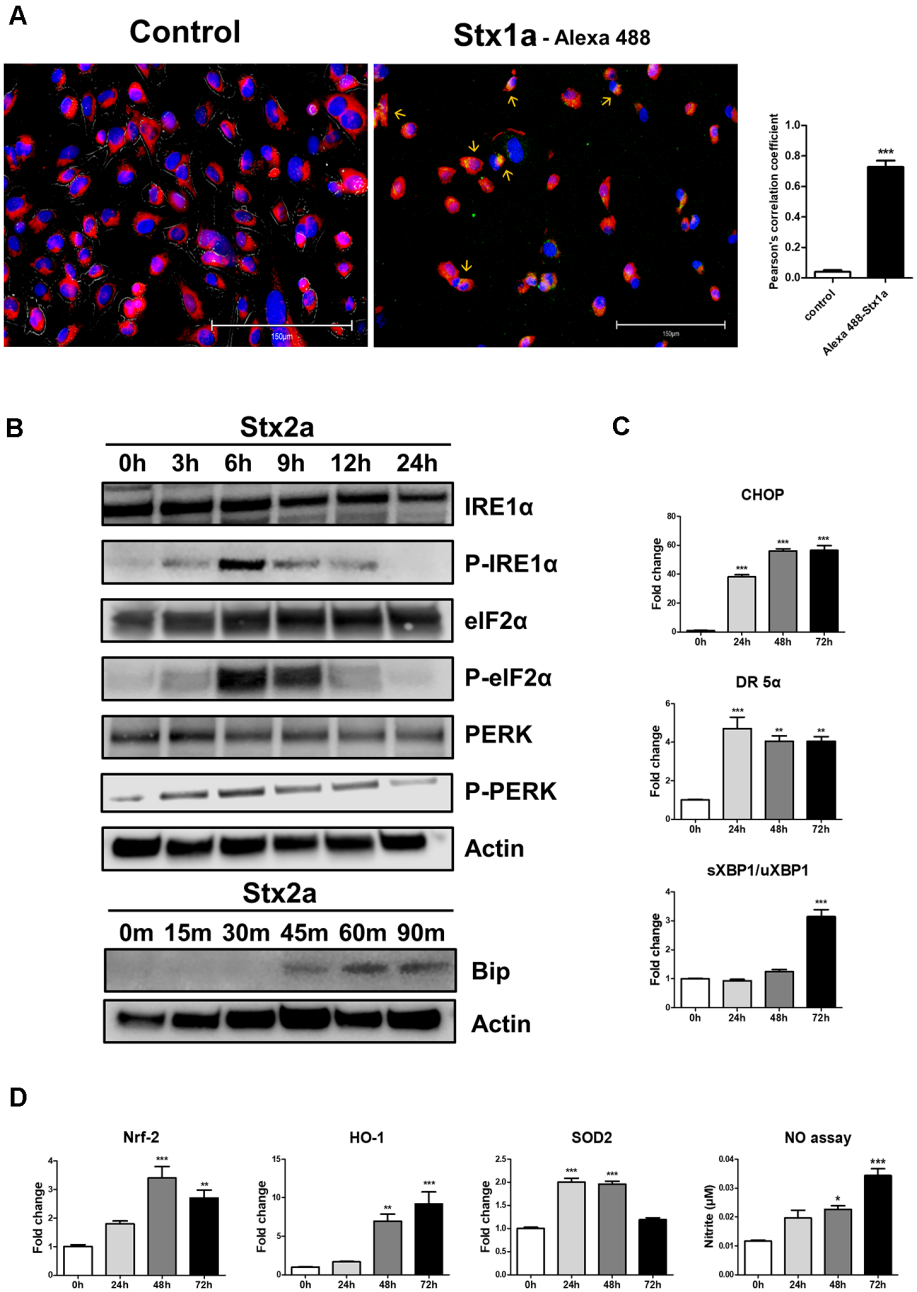
Treatment with Stx1a or Stx2a induces ER stress and ROS generation following intracellular translocation of toxins to the ER in hCMEC/D3 cells. (**A**) hCMEC/D3 cells were seeded in 6-well plates (5.0 × 10^5^ cells/well). After washing with culture medium, cells were fixed and nuclei were stained with DAPI reagent. Representative DAPI-positive cells were visualized by fluorescence microscopy. To detect Stx translocation to the ER, cells were stimulated with complete growth medium containing 50 nM ER-tracker (red) live cell staining dye 2 h after treatment with Alexa Fluor 488-conjugated Stx1a (100 ng/ml). After washing, cells were captured using a fluorescence microscope EVOS M5000. Yellow fluorescence indicates co-localization of Stx1a with the ER marker. The scale bars represent 150 μm. The bar graph represents the mean ± SEM of the Pearson's correlation coefficient for the co-localization of Stx1-Alexa Fluor 488/ER tracker. At least 30 cells per condition were analyzed. Asterisks indicate statistically significant differences between the control and Stx1-treated groups. *** = *p* < 0.001; (**B**) hCMEC/D3 cells were stimulated with Stx2a (10 ng/ml) for 0, 3, 6, 9, 12 and 24 h. After washing, cells were lysed at the indicated time points, and the presence of activated ER stress sensors and downstream targets in the cell lysates was determined by Western blotting. Untreated cells served as controls, and β-actin was used as a loading control; (**C**) hCMEC/D3 cells were treated as described above, and CHOP and DR5, spliced XBP1/unspliced XBP1 mRNA expression was measured by RT-qPCR and normalized to glyceraldehyde-3-phosphate dehydrogenase (GAPDH); (**D**) hCMEC/D3 cells were stimulated with Stx2a (10 ng/ml) for 0, 24, 48, and 72 h. At the indicated time points, cells were lysed, and the expression of ROS-related markers (Nrf-2, HO-1, SOD-2, NO2) in cell lysates was measured by RT-qPCR and normalized using GAPDH. Asterisks indicate significant differences between control cell values and Stxs-treated cells (Panels C and D) at each time point. * = *p* < 0.05; ** = *p* < 0.01; *** =*p* < 0.001. Data are shown as mean ± SEM from three independent experiments. *p < 0.05, **p < 0.01, ***p < 0.001 vs. control.

To visualize Stxs internalization in hCMEC/D3 cells [7], we examined intracellular trafficking using immunofluorescence microscopy. To identify the cellular pathway, hCMEC/D3 cells were treated with Alexa Fluor 488-conjugated Stx1a. Cell translocation was observed after 2 h (Fig. 2A). Fluorescent signals were observed to coalesce around the nucleus, indicating intracellular translocation of the Stxs. Notably, Alexa Fluor 488-conjugated Stx1a was co-localized with an ER-specific fluorescent marker, resulting in a yellow fluorescence signal in the image, indicating retrograde translocation of the Stxs to the ER. We then examined ER stress and increased ROS in hCMEC/D3 cells exposed to Stxs. Inositol-requiring enzyme 1α (IRE1α) and eukaryotic translation initiation factor-2α (eIF2α) were confirmed to be phosphorylated to the highest level at 6 h, while protein kinase RNA-like endoplasmic reticulum kinase (PERK) was activated at 3 h and binding immunoglobulin protein (BiP) at 45 min, respectively (Fig. 2B). In addition, C/EBP homologous protein (CHOP) and death receptor 5 (DR5), ER stress-related factors that induce apoptosis, were significantly increased after Stx2a treatment compared to the control (Fig. 2C). Concordantly, oxidative stress-responsive proteins were upregulated, including Nuclear factor erythroid 2-related factor 2 (Nrf-2), Heme Oxygenase-1 (HO-1), Superoxide dismutase 2 (SOD-2), and nitrosative signals (NO_2_), indicating a Stx2a-dependent elevation in ROS. These results suggest that Stx2a is associated with the induction of ER stress and the rise of ROS synthesis in hCMEC/D3 cells.

### Stxs Activate MAPK Signaling and Upregulate Inflammatory Cytokines in hCMEC/D3 Cells

**Fig. 3:**
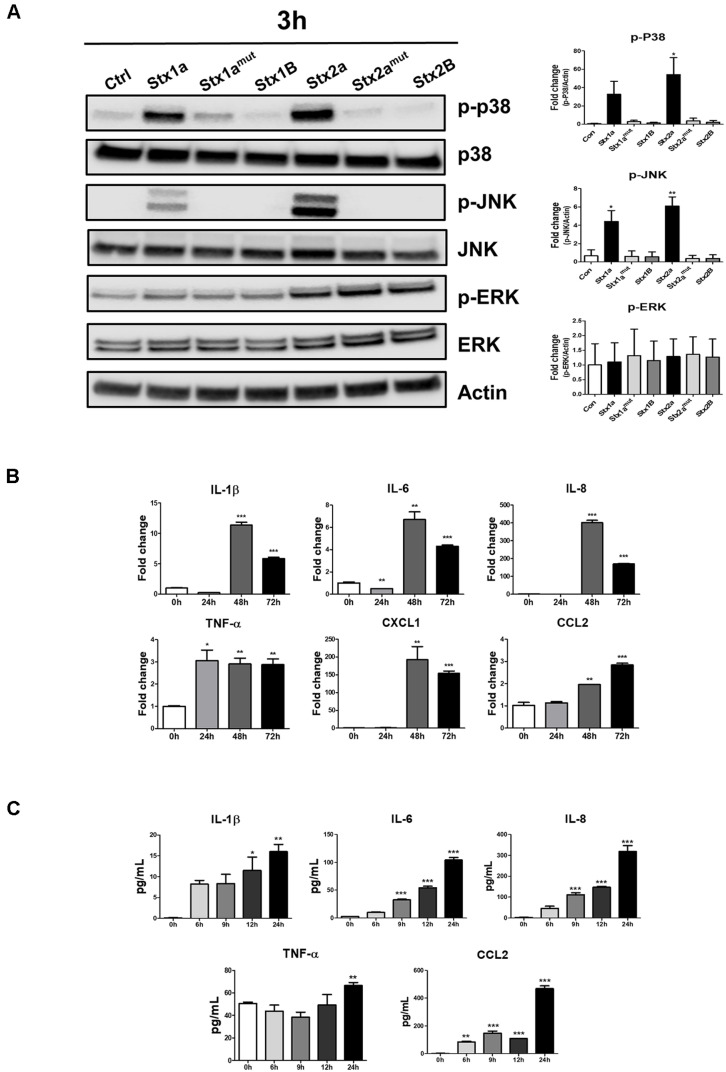
MAPK activation and induction of inflammatory cytokines following Stx1a and Stx2a treatment in hCMEC/D3 cells. (**A**) hCMEC/D cells were seeded in 6-well plates at a density of 5.0 × 10^5^ cells/well and treated with Stx1a (100 ng/ml), Stx1a^mut^ (100 ng/ml), Stx1B (100 ng/ml), Stx2a (10 ng/ml), Stx2a^mut^ (10 ng/ml), or Stx2B (10 ng/ml) for 3 h. After 3 h, cells were washed and cell lysates were collected. Phosphorylation of p38, JNK, and ERK were assessed by Western blotting. β-Actin was used as a loading control. The graphs show the mean ± SEM of band densities normalized by the division of β-Actin band densities and compared to untreated control cell values (*right panel*). Statistical analyses of densitometric scans from at least three independent experiments are shown; (**B**) hCMEC/D3 cells were seeded in 6-well plates at a total cell density of approximately 5.0 × 10^5^ cells/well and treated with Stxs for 0, 24, 48, and 72 h. At each time point, cells were lysed and mRNA expression levels of inflammatory cytokines (IL-1β, IL-6, IL-8, TNF-α, CXCL1, and CCL2) were measured by RT-qPCR and normalized to GAPDH; (**C**) Under the same conditions, supernatants were collected and protein levels of IL-1β, IL-6, IL-8, TNF-α and CCL2 were quantified by ELISA using kit standards. Data are presented as mean ± SEM from three independent experiments. Asterisks indicate significant differences between control and Stx-treated cells (Panels B, C) at the indicated time points. *p < 0.05; **p < 0.01; ***p < 0.001.

ER stress response activated by Stxs induces the MAPK signaling pathway [21, 22]. Therefore, we examined whether Stxs activate MAPK signaling in hCMEC/D3 cells. When the cells were treated with Stx1a and Stx2a, robust phosphorylation of p38 and c-Jun N-terminal kinase (JNK) was observed at 90 to 180 min (Fig. S2), whereas no phosphorylation was observed when treated with Stx1a^mut^, Stx2a^mut^, and the B subunit. In contrast, extracellular signal–regulated kinase (ERK) was phosphorylated in the B subunit and Stx1a^mut^, Stx2a^mut^ (Fig. 3A), suggesting that the apoptosis-linked MAPK activity in this context is dominated by p38/JNK rather than ERK. To confirm that Stx2a induces the expression of inflammatory cytokines in hCMEC/D3 cells, we measured the mRNA and protein levels of representative inflammatory cytokines, such as IL-1β, IL-6, TNF-α, and IL-8. The expression of inflammatory cytokines significantly increased at all time points compared to the control group (Fig. 3B and 3C). Therefore, the exposure to Stx activates a MAPK pathway centered on p38/JNK, which aligns with the induction of pro-inflammatory cytokines in hCMEC/D3 cells.

### Stxs Impair the Integrity of Tight Junctions and Increase Endothelial Permeability

**Fig. 4:**
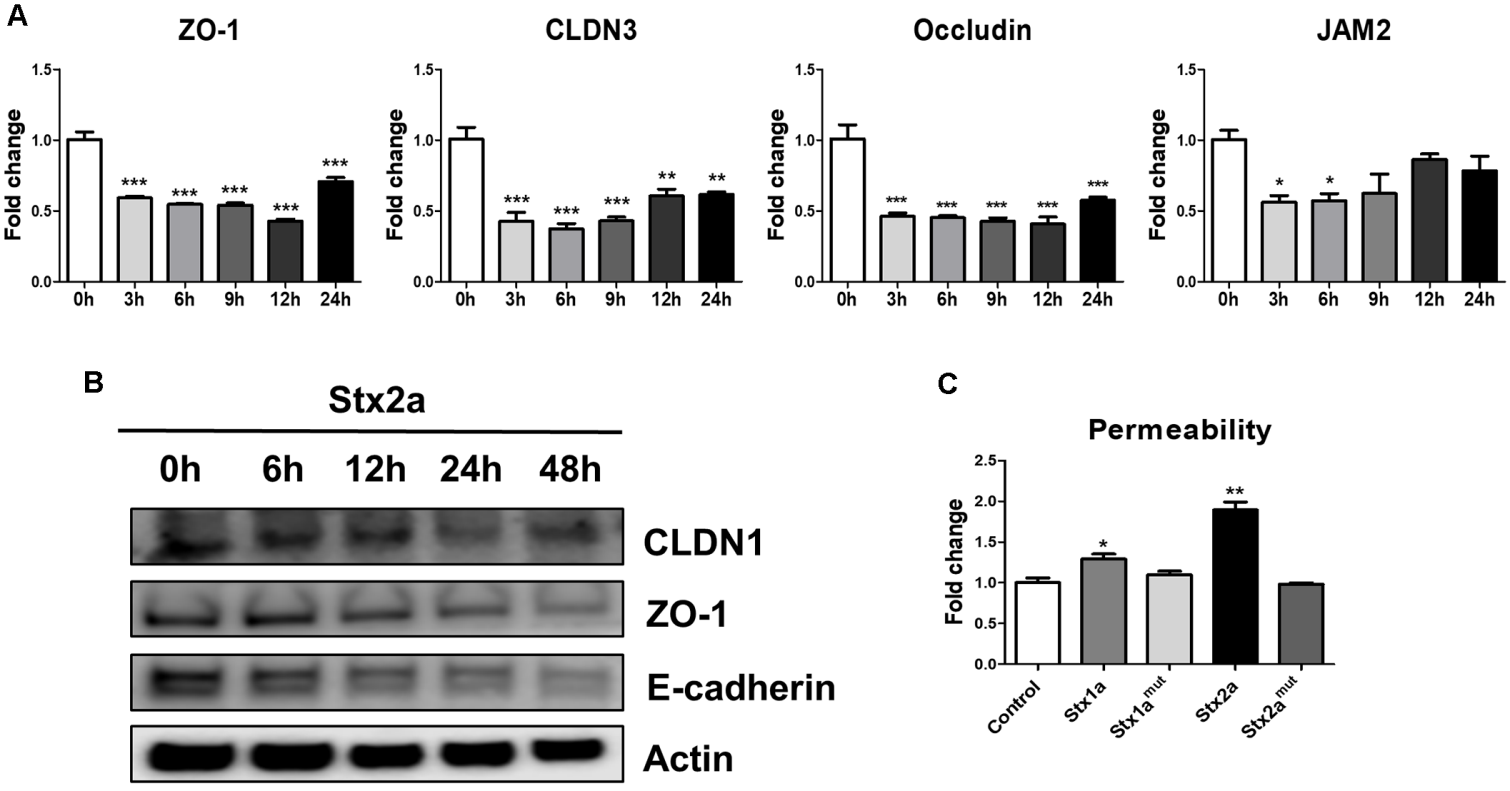
Stx exposure in hCMEC/D3 cells results in a decrease in tight junction proteins and an increase in permeability. (**A**) hCMEC/D3 cells were seeded in 6-well plates at a total cell density of approximately 5.0 × 10^5^ cells/well and treated with Stxs for 0, 3, 6, 9, 12, and 24 h. At each time point, cells were lysed, and mRNA expression of ZO-1, CLDN3, Occludin and JAM2 was measured by RT-qPCR. Expression levels were normalized to GAPDH; (**B**) hCMEC/D3 cells were seeded as above and treated with Stx2a for 0, 6, 12, 24, and 48 h. At each time point, cells were lysed, and the tight junction proteins CLDN1, ZO-1, and Ecadherin were analyzed by Western blotting. β-Actin was used as a loading control; (**C**) hCMEC/D3 cells were seeded in the insert wells of Trans-well plates at 3.0 × 10^5^ cells/well. Cells were treated with Stx1a (100 ng/ml), Stx1a^mut^ (100 ng/ml), Stx2a (10 ng/ml), or Stx2a^mut^ (10 ng/ml) for 24 h. Fluorescein-conjugated 45kDa ovalbumin was added to the toxin-treated insert wells, and supernatants were collected from the lower chamber after 1 h. Cell permeability was evaluated by measuring the fluorescence of ovalbumin translocated from the insert wells to the lower chamber using a microplate reader. Asterisks indicate significant differences between the control cell values and the Stxs-treated cells (Panels A, C) at each time point. * = *p* < 0.05; ** =*p* < 0.01; ***=*p* < 0.001.

TJs between brain endothelial cells assemble into intercellular complexes that strictly limit the transport and diffusion of specific substances across the BBB, thereby serving as its essential barrier [13, 23-25]. Representative TJ markers include Zonula Occludens-1 (ZO-1), Claudin3 (CLDN3), Occludin, and Junctional Adhesion Molecule 2 (JAM2). In this study, we confirmed that the mRNA levels of TJs were significantly reduced beginning at 3 h after Stxs treatment (Fig. 4A). In particular, the downward trends of ZO-1, CLDN3, and occludin spanned 9–12 h, that of JAM2 progressed until ~9 h with subsequent restoration in part. Followed at the protein level, CLDN1, E-cadherin (a component of the adherens junction), and ZO-1, known as markers of endothelial cells, declined over time gradually decreased over time (Fig. 4B). Functionally, permeability in hCMEC/D3 monolayers treated for 24 h in Stx1a or Stx2a was elevated to ovalbumin, whereas no increase in permeability was discernible in control or in enzymatically inactive mutants Stx1a^mut^, Stx2a^mut^ (Fig. 4C). Accordingly, Stx exposure reduces TJ protein expression and increases paracellular flux in human cerebral endothelium.

### Caspase-Mediated Apoptosis and ROS Propel Stxs-Induced Cell Damage and Tight Junction Disruption

**Fig. 5:**
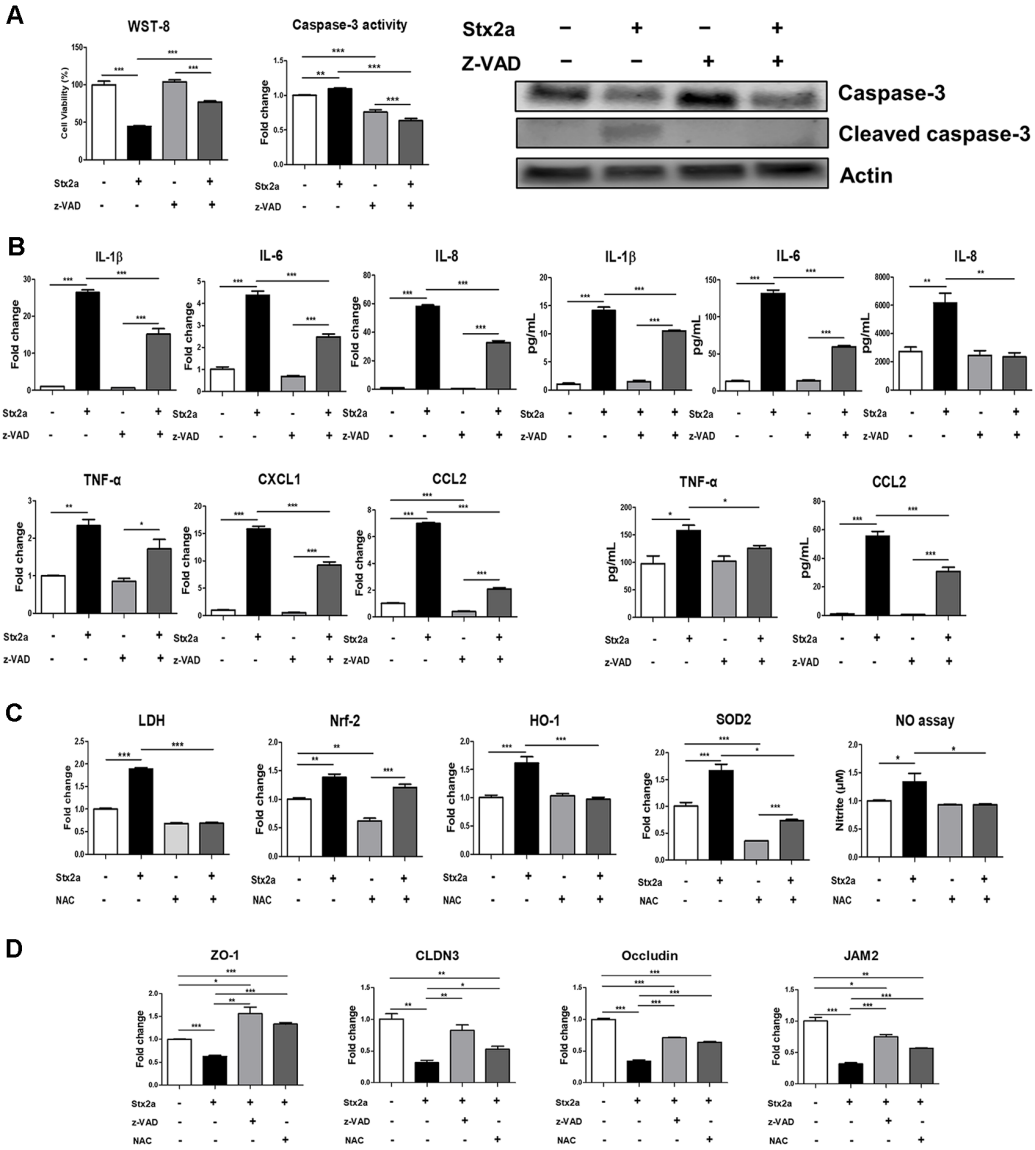
z-VAD and NAC attenuate Stx2a-Induced Apoptosis, Inflammation, and Tight Junction Disruption in hCMEC/D3 Cells. (**A**) hCMEC/ D cells were seeded in 6-well plates. Cells were pretreated with z-VAD (20 μM) for 1 h before Stx2a exposure and divided into the following groups: untreated control, Stx2a-treated, z-VAD only, and Stx2a + z-VAD pre-treated. After 24 h of Stx2a treatment, cell viability was assessed using the WST-8 assay (*left panel*). Subsequently, Western blot analysis showed that the total levels of caspase-3/7 in the cell lysates were decreased only in the Stx2a-treated group. Protein samples were prepared using anti-caspase-3, anti-cleaved caspase-3, and anti-β-Actin antibodies (*right panel*). β-Actin was used as a control for equal protein loading; (**B**) hCMEC/D3 cells were seeded as described above and treated with z-VAD 1 h before Stx2a treatment. Cell lysates were harvested from the following groups: untreated control, Stx2a-treated, z-VAD only, and Stx2a + z-VAD pre-treated. Culture supernatants were collected, and the levels of secreted IL-1β, IL-6, IL-8, TNF-α and CCL2 were measured using ELISA (*left panel*). Total RNA was isolated from cell lysates, and mRNA expression levels of IL-1β, IL-6, IL-8, TNF-α, CXCL1, and CCL2 were quantified using RT-qPCR (*right panel*). RNA expression levels were normalized using GAPDH; (**C**) hCMEC/D3 cells were seeded in 6-well plates at a total cell density of 5.0 × 10^5^ cells/well and treated with NAC (5μM) 1 h before Stx2a exposure. The experimental groups included control, Stx2a-treated, NAC only, and Stx2a + NAC pre-treated. After 24 h of Stx2a treatment, the cytotoxicity level was measured by LDH assay using the cell culture supernatant. mRNA expression levels of Nrf-2, HO-1, and SOD-2 were measured by RT-qPCR and normalized to GAPDH, and the degree of NO expression was measured by NO assay; (**D**) hCMEC/D3 cells were seeded in 6-well plates at a density of 5.0 × 10^5^ cells/well and treated with z-VAD and NAC 1 h before Stx2a treatment. Cell lysates were harvested from the following groups: control, Stx2a-treated, z-VAD + Stx2a, and NAC + Stx2a groups. After 24 h of Stx2 treatment, washed cell lysates were harvested, and the mRNA expression of ZO-1, CLDN3, Occludin and JAM2 were measured using RT-qPCR. RNA expression levels were normalized using GAPDH. Asterisks indicate significant differences compared to the control group, Stx-treated group, or between monotherapy vs. combination therapy (e.g., z-VAD alone vs. z-VAD+Stx2a) (Panels A, B) and compared to the control group, Stx-treated group, or between monotherapy vs. pre-treatment therapy (*e.g.*, NAC alone vs. NAC+Stx2a) (Panel C). Significant differences are indicated between the control and cells treated with Stx, Stx+z-VAD, or Stx+NAC (Panel D).* = *p* < 0.05; ** = *p* < 0.01; *** = *p* < 0.001.

To identify whether apoptosis and oxidative stress engage in Stx damage, we pretreated hCMEC/D3 cells with z-VAD (pan-caspase inhibitor) or N-acetylcysteine (NAC; ROS scavenger) and tested viability, cytokine expression, and junctional protein expression. Pretreatment with z-VAD substantially improved viability in comparison with Stx2a alone, reducing viability (Fig. 5A). Our dependence on caspases was defined by cleaved caspase-3 immunoblotting: there was evident cleaved caspase-3 in Stx2a-treated cultures, no evident signal in control or control+z-VAD cultures, and no evident signal in Stx2a+z-VAD cultures (Fig. 5A). To assess inflammatory outputs, we tested levels of IL-1β, IL-6, IL-8, TNF-α, CXCL1, and CCL2 at mRNA/protein levels; all were upregulated with Stx2a alone and downregulated by z-VAD (Fig. 5B). To define the role of the oxidative stress, NAC pretreatment reduced LDH release in comparison with Stx2a alone (Fig. 5C). Stx2a robustly upregulated markers of oxidative stress (Nrf-2, HO-1, SOD-2) as well as nitrosative signals (NO2), and NAC reduced these readouts, confirming toxin-induced ROS/RNS accumulation. Finally, in order to correlate these pathways with loss of barrier, we examined the expression of ZO-1, CLDNs, occludin, and JAM2 after pretreatment with z-VAD or NAC. Stx2a reduced TJ proteins by itself, while pretreatment with z-VAD or NAC reversed their expression partially, confirming that caspase-dependent apoptosis and ROS signaling engage to stimulate.

### Stxs Induce Caspase-Dependent Apoptosis and Inflammatory Responses in an hCMEC/D3-A172 Co-Culture Model of the BBB

**Fig. 6:**
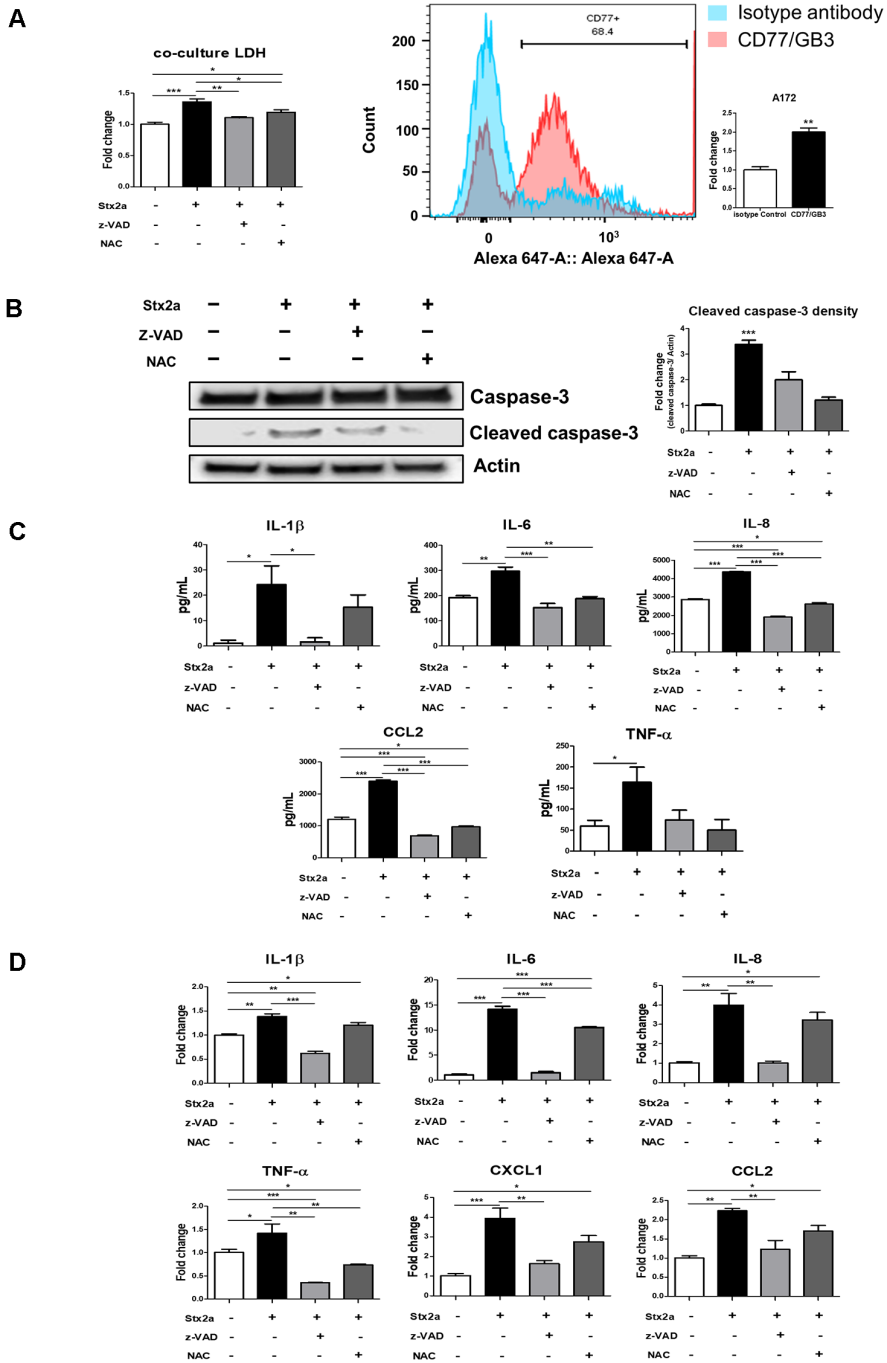
Stxs induced caspase- and ROS-dependent inflammation and cytotoxicity in a BBB co-culture model. (**A**) hCMEC/D3 cells were seeded in the insert wells of Trans-well plates at 3.0 × 10^5^ cells/well, and A172 cells were seeded in the bottom wells of Trans-well plates at 5.0 × 10^5^ cells/well. The insert wells containing hCMEC/D3 cells were stimulated with Stx2a (10 ng/ml) for 24 h. After 24 h, the supernatants were collected and cytotoxicity in A172 cells was assessed by LDH *assay* (*left panel*). A representative flow-cytometry readout of Gb3 in A172 astrocytes (*right panel*). (**B**) Subsequently, the A172 cells in the bottom wells were washed, and the cell lysates were harvested and the protein levels of caspase-3 and cleaved caspase-3 were determined by Western blotting. Anti-caspase-3, anticleaved caspase-3, and anti-β-Actin antibodies were used for protein samples. β-Actin served as a loading control (**C-D**) Culture supernatants of A172 cells seeded in the same manner as above were harvested, and the secretion levels of IL-1β, IL-6, IL-8, TNF-α, and CCL2 were determined using ELISA. Subsequently, the mRNA expression levels of IL-1β, IL-6, IL-8, TNF-α, CXCL1 and CCL2 were assessed by using RT-qPCR in cell lysates. RNA expression levels were normalized using GAPDH. Asterisks indicate significant differences between the control group and cells treated with Stx, Stx+z-VAD, and Stx+NAC (Panels A, C, D). * = *p* < 0.05; ** = *p* < 0.01; ***= *p* < 0.001.

A transwell co-culture *in vitro* model was established using the human brain endothelial cell line, hCMEC/D3, and the human astrocyte cell line, A172, to more closely resemble an efficient and functional microenvironment of the BBB [18, 19]. The setup allows the indirect or paracrine effects of molecules, including toxins and inflammatory mediators, to be transmitted between the two cell types [26]. After exposure of hCMEC/D3 in the insert to Stx, A172 cells in the basal compartment showed impaired viability relative to untreated controls (Fig. 6A, left panel). Flow cytometry shows a clear rightward fluorescence shift for CD77/Gb3 (red) relative to the isotype control (blue), with the gate indicating ~69% CD77^+^ cells. The accompanying bar graph quantifies a significant increase in signal (fold-change), confirming surface Gb3 expression on A172 astrocytes (Fig. 6A, right panel). Although the A172 cells were not exposed directly to Stx, the presence of Gb3 accompanies their inherent susceptibility as well as provides a mechanism for the paracrine injury evident in the co-culture. In parallel, cleavage of caspase-3 was readily apparent in A172 after challenge of the overlying endothelium by Stx, yet was attenuated by z-VAD or NAC, reflecting paracrine, caspase-dependent apoptosis with a facet of ROS (Fig. 6B). In concordance, A172 showed higher levels of mRNA/protein expression of the inflammatory/apoptotic mediators IL-1β, IL-6, IL-8, TNF-α, and CCL2 in the Stx2a-only condition, yet pretreatment with z-VAD or NAC diminished these reactions to the cytokines (Fig. 6C and 6D). These observations suggest that Stx-injured endothelium delivers ROS- and caspase-linked inflammatory/apoptotic signals to the astrocytes, consistent with endothelial-to-astroglial crosstalk as an explanation for neurovascular damage mediated by the toxin.

## Discussion

While acute renal failure is the signature of Stx-mediated HUS, the pathobiology at the blood–brain barrier (BBB) remains relatively undefined. Here, we delineated how human brain microvascular endothelial cells (hCMEC/D3) respond to Shiga toxins and demonstrated that TJ architecture, a determining factor in BBB function, is disrupted following exposure to the toxin. Importantly, we report that Stx1a and Stx2a induce apoptosis in hCMEC/D3 cells, confirming endothelial susceptibility to Stx cytotoxicity and revealing time-dependent morphological deterioration consistent with prior reports [27, 28]. Cytotoxicity was verified by phase-contrast microscopy, WST assays, and caspase-3/7 activity, and mechanistically attributed to toxin internalization, ER-stress and MAPK activation, and the release of pro-inflammatory cytokines [29-31]. Congruent with the paradigm that Gb3 expression confers Stx susceptibility [2, 32], hCMEC/D3 cells exhibited highly extensive availability of cell surface receptor, as indicated by FACS analysis of A4GALT (Gb3 synthase) and Gb3. Fluorescent recombinant Stx were efficiently internalized and retrogradely trafficked to the ER within 60 min, in favor of canonical Gb3-dependent uptake and retrograde routing (Fig. 2). Once delivered to the ER, Stxs activated the unfolded protein response (UPR), with induction of BiP, CHOP, XBP1, PERK, and IRE1α, and concomitantly increased intracellular ROS (Fig. 2). Because UPR and oxidative stress often intersect with stress-activated kinase cascades, we interrogated the MAPK axis (Fig. 3): Stx exposure robustly phosphorylated p38 and JNK, whereas an enzymatically inactive Stx mutant (Stx1a^mut^, Stx2a^mut^) and isolated B-subunit did not. Conversely, ERK phosphorylation was observed with the B-subunit and with Stx mutant (Stx1a^mut^, Stx2a^mut^), suggesting that the apoptosis- and inflammation-linked MAPK signal in this model follows p38/JNK rather than ERK [10]. Together, these data support a model in which Stx activation induces ER stress and ROS accumulation, driving activation of p38/JNK, cytokine induction, caspase activation, and apoptosis in brain endothelial cells. Functionally, these events compromise BBB structure and function. TJs, comprised of claudins, occludin, junctional adhesion molecules, and scaffold proteins such as ZO-1, govern paracellular barrier selectivity and endothelial polarity [33, 34]. In our model, CLDN3, occludin, JAM2, and ZO-1 were significantly reduced following Stx exposure (Fig. 4), in line with TJ destabilization and barrier failure. CLDN3 is a reported BBB-endothelial constituent and is stably expressed in hCMEC/D3 [25], with ZO-1 scaffolds multiple TJ/adherens components to regulate cell–cell adhesion [35]. In keeping with the structural loss, we observed elevated paracellular permeability, including with Stx vs Stx1a^mut^, Stx2a^mut^ treatment (Fig. 4C) [36]. Notably, co-treatment with z-VAD (pan-caspase inhibitor) lowered cytotoxicity, preserved TJ proteins, and dampened cytokine production, indicating that caspase-dependent apoptosis is a principal executioner of the observed loss of barrier. Similarly, N-acetylcysteine (NAC) pretreatment mitigated ROS and reduced subsequent apoptotic signaling, supporting a ROS→apoptosis axis in the toxin-injured endothelium [37]. Collectively, these data indicate that Stx-induced inflammatory and apoptotic programs are major drivers of the observed permeability increase. Noting that BBB integrity emerges from neurovascular unit crosstalk, we then employed an *in vitro* transwell co-culture model of hCMEC/D3 endothelium in the presence of human astrocytes (A172) [18, 19]. Strikingly, astrocytes not directly exposed to Stx nonetheless exhibited cytotoxicity, cytokine induction, and increased cleaved caspase-3, indicating that paracrine endothelial–astroglial signaling can propagate injury across the barrier interface (Fig. 6). Although A172 cells were not directly exposed to Stx, the presence of Gb3 supports their intrinsic susceptibility and provides a mechanistic basis for the paracrine injury observed in the co-culture (*i.e.*, once toxin or toxin-induced mediators traverse the endothelial layer, astrocytes may possess the receptor to engage downstream responses). As shown in Fig. 6D, pharmacologic rescue at the endothelial compartment with pan-caspase inhibition (z-VAD) or ROS scavenging (NAC) partially preserved astrocyte viability and reduced caspase activation and cytokine release, suggesting that endothelial caspase activation and oxidative stress are essential upstream initiators of astroglial damage caused by Stx2a. These observations align with a model in which endothelial injury serves as a nexus that amplifies inflammatory/apoptotic cues to neighboring glia, presumably through the action of soluble factors and damage-associated signals, thereby potentially further expanding the impact of neurovascular damage [8, 38].

In conclusion, our findings demonstrate that hCMEC/D3 endothelium is highly susceptible to Stx1a/Stx2a, undergoing time-dependent apoptosis via ER-stress and stress-kinase pathways, with resultant secondary ROS accumulation and pro-inflammatory signaling. This apoptotic program reduces TJ components (CLDN3, ZO-1, occludin, JAM2) and increases paracellular permeability. Pharmacologic caspase blockade (z-VAD) and ROS scavenging (NAC) maintain viability, lessen cytokine production, and stabilize TJ expression, indicating that apoptosis in endothelial cells may promote BBB compromise. We note the following limitations: the *in vivo* BBB consists of endothelium, pericytes, and astrocytes, and our co-culture lacked pericytes; additionally, *in vivo* shear stress and full immune components were not modeled [12, 39]. Nonetheless, by integrating receptor-dependent toxin entry, retrograde ER trafficking, UPR/ROS–MAPK signaling, and apoptotic TJ loss with endothelial–astroglial crosstalk, our data provide a coherent mechanistic scheme linking circulating Stx to cerebrovascular injury and neurologic manifestations, in particular stroke, seizures, and encephalopathy, in atypical HUS [8, 40, 41]. Our findings suggest a pathophysiological sequence in which Stx is transported via the bloodstream to the cerebral microvessels, binds Gb3 on brain endothelium, undergoes retrograde entry to the ER, triggers ROS-coupled apoptotic and inflammatory pathways, and structurally disrupts the BBB, thereby facilitating CNS complications. Further studies with the inclusion of pericytes, immune components, and physiological flow, alongside targeted modulation of ER-stress/ROS–caspase cascades, will be helpful in refining therapeutic strategies for preventing Stx-mediated neurovascular injury.

## Supplemental Materials

Supplementary data for this paper are available on-line only at http://jmb.or.kr.



## Figures and Tables

**Table 1:** RT-qPCR primers sequence.

Gene	Type	Sequence
DR5	Forward	AAGACCCTTGTGCTCGTTGT
	Reverse	GGAGCTAGGTCTTGTTGGGT
CHOP	Forward	TAGGGGACATGTGTGAGCATGA
	Reverse	TCACGGCAAAGAGATCGGAGA
GAPDH	Forward	GCACCGTC AAGGCTGAGAAC
	Reverse	TGGTGAAGACGCCAGTGGA
Nrf-2	Forward	GCAGACATTCCCGTTTGTAGA
	Reverse	AGGTGACTGAGCCTGATTAGTA
SOD-2	Forward	AATCAGGATCCACTGCAAGG
	Reverse	TAAGCGTGCTCCCACACAT
sXBP1	Forward	TGAGTCCGCAGCAGGTGCA
	Reverse	CTGGGTCCTTCTGGGTAGACCTC
uXBP1	Forward	CTCATGGCCTTGTAGTTGAGA
	Reverse	AGGGCATTTGAAGAACATGAC
HO-1	Forward	ATGGCCTCCCTGTACCACATC
	Reverse	TGTTGCGCTCAATCTCCTCCT
IL-1β	Forward	GGACAGGATATGGAGCAACAA
	Reverse	CCCAAGGCCACAGGTATTT
IL-6	Forward	GATGAGTACAAAAGTCCTGATCCA
	Reverse	CTGCAGCCACTGGTTCTGT
CCL2	Forward	GTCTCTGCCGCCCTTCTGTG
	Reverse	AGGTGACTGGGGCATTGATTG
TNF-α	Forward	AGCCCATGTTGTAGCAAACC
	Reverse	TCTCAGCTCCACGCCATT
CXCL1	Forward	CCCAAGAACATCCAAAGTGTGA
	Reverse	CAAGCTTTCCGCCCATTCT
IL-8	Forward	GGTATCCAAGAATCAGTGAAGA
	Reverse	CTACAACAGACCCACACAATA
ZO-1	Forward	CCCCACTCTGAAAATGAGGA
	Reverse	GGGAACAACATACAGTGACGC
CLDN3	Forward	AGCAGCGAGTCGTACACCTT
	Reverse	AACATCATCACGTCGCAGAA
Occludin	Forward	TGTGATGAGCTGGAGGAGGA
	Reverse	TTCCTGTAGGCCAGTGTCAAA
JAM2	Forward	TCTTCTTTGGGGTTTTGCAG
	Reverse	TACCTGGTGGTCGCCCT
