# Supplementary material for: Shiga Toxin Induces Apoptosis via ROS–Caspase Activation in Human Cerebral Endothelial Cell Line hCMEC/D3 and Astrocyte Co-Culture
Source: J Microbiol Biotechnol. 2026 Jan 8;36:e2512006. doi: 10.4014/jmb.2512.12006 (PMC12790988; doi:10.4014/jmb.2512.12006)
Supplement: Supplementary file 1 [file jmb-36-e2512006-supple.pdf]

J. Microbiol. Biotechnol. 2026. 36: e2512006

https://doi.org/10.4014/jmb.2512.12006

Supplementary Figures

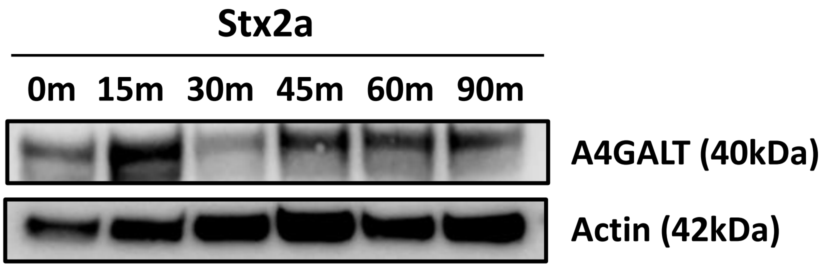

Fig. S1. Western blot analysis of A4GALT, a Gb3 synthase, following Stx2a treatment.

Western blot analysis of A4GALT, a Gb3 synthase, was performed at 0, 15, 30, 45, and 90 min following Stx2a treatment. A4GALT protein was detectable at all time points, with expression peaking at 15 min. A transient decrease in expression was observed at 30 min, followed by sustained levels from 45 to 90 min. β-Actin was used as a control for equal protein loading. The results are a representative experiment obtained from three independent experiments.

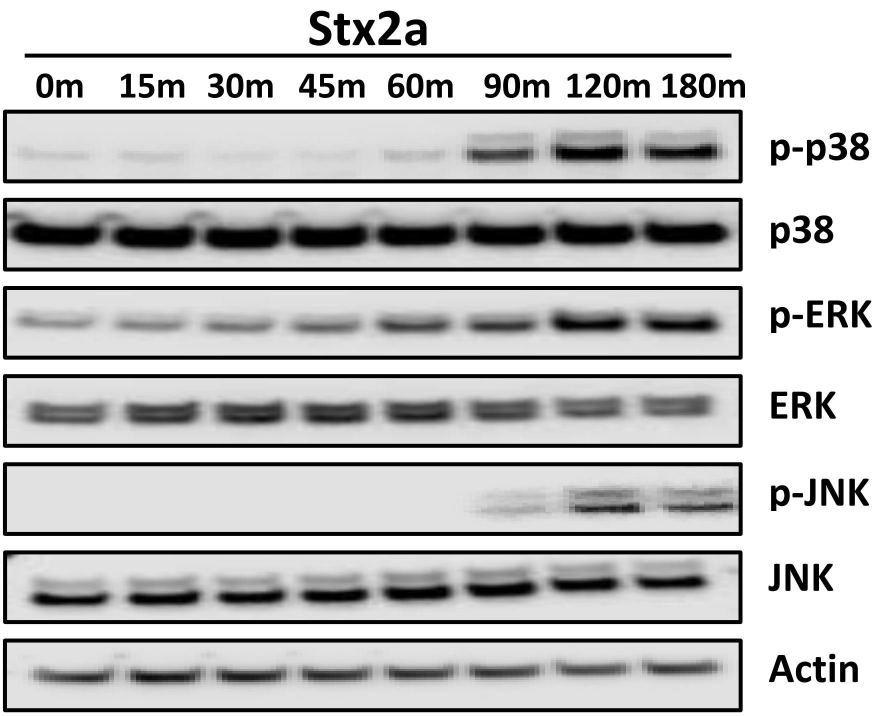

Fig. S2. Time-dependent activation of MAPKs following Stx2a treatment.

Western blot analysis was performed to assess the activation of MAPKs (p38, ERK, and JNK) from 0 to 180 min after Stx2a treatment. Total p38, ERK, and JNK were detectable at all time points. Phosphorylated p38 (p-p38) and phosphorylated JNK (p-JNK) were first observed at 90 minutes, with maximal expression at 120 min. Phosphorylated ERK (p-ERK) was detectable throughout the time course. β-Actin was used as a loading control. Representative blots from three independent experiments are shown.
